# Annotations

**Published:** 1908-03-21

**Authors:** 


### ANNOTATIONS.

### The Sleeping Sickness Commission.

Although the second International Sleeping Sickness Commission, which has just been held in London, has come to an abortive end, owing to the absence of unanimity of the delegates upon certain important points, there is no reason to suppose that the Conference has achieved nothing. Lord Fitzmaurice, the President, is quite certain that the papers and discussions and the exchange of opinions, which would never have taken place but for this meeting of international experts, constitute an important record in themselves, and will help towards a future unanimity upon controverted points. But the Congress, even if it should lead to no immediate practical result so far as the problem of sleeping sickness is concerned, has also been an important step towards international co-operation for the common good of humanity. Owing to the inability of one or two of the delegates to agree with the proposals of the majority, concerning the establishment of a central bureau and other matters, the draft convention has not been accepted. Nevertheless, the progress of the investigation of sleeping sickness has been stimulated by this Conference, and what is even more important, the possibility of the advancement of medical science by the combined action of representatives of the leading powers has been demonstrated. The universality of science and of medicine is gradually being recognised, and the recent Congress has achieved something in this direction if in no other. The medicine of the future will be largely preventive, and the progress of prophylactic medicine will mainly depend upon international co-operation, and it is satisfactory to know that the organisation of the Sleeping Sickness Commission, in itself a practical application of these two principles, was due to a great extent to the initiative of the British Government and of British medical men.

### Socialism, Cancer, and Advertisement.

A curious publication has lately reached us. It is the second number of a penny periodical entitled "The Stormy Petrel: for Justice and Liberty, for God, our Homes and Country: a Week-end Magazine for Thinking Men and Women, edited by John Shaw, M.D.(Lond.)." After this suggestive title we were not surprised to find that the contents were mainly devoted to the combined vindication of Socialism and the treatment of cancer without operation. At the end of the editorial article is a prominent advertisement of Dr. Shaw's works on cancer, already too well-known to require particularisation at our hands. Then comes an inflammatory reprint from *The Clarion*, and after that an attack upon British judges entitled "John Bull's Cancer." In fact, there is a great deal about cancer, one way or another, in Dr. Shaw's latest departure. "Why I am a Socialist," an unsigned editorial contribution is followed by an advertisement of Dr. Shaw's work on "Medical Priestcraft," an attack on the medical profession. Later we find a further long contribution from the same pen upon "Cancer: the Quicksands of Operation." But the cream of the paper really lies in the two back pages of the cover, the first being another advertisement of the editor's speciality, signed with his name and professional address, and the second being an advertisement of the editor's five-shilling Treatment of Influenza by post. It may interest the General Medical Council, as well as the body of the profession, to learn that Dr. John Shaw, in addition to being the editor of this periodical, is also its proprietor and publisher. We cannot honestly wish him success.

### Cheap Food for School Children.

Now that free meals are being given to school children under the Education Department of the County Council it becomes a question of general interest to find the cheapest rate at which good wholesome food can be provided. It must be confessed that we English are extravagant in our food beyond any nation on the earth, except the Americans. In both cases this extravagance springs from the general high standard of prosperity enjoyed by both nations. But when people are fed from public funds it is only right to consider it there are to be found substitutes for our favourite meat and puddings which are equally nourishing, not unpalatable, and cheaper. Such substitutes are to be found in many cereals, in the pulses, and in nuts and many vegetables. Most of these substances require longer and more careful cooking than meat in order to be palatable, and this is a quite comprehensible reason why they are not so much used as they might be in private households. Coals cost money as well as food; but when children are to be fed by the hundred the cheapness of the prime cost of the food far outweighs the cost of cooking. Believing this, the Bread and Food Reform League propose to appeal to the Education Department of the London County Council asking the department to give a place in their menus to dishes formed of the cheap and nourishing foods we have mentioned. They do not press for an entirely vegetarian diet, but suggest that soups, stews, and puddings made of vegetables which have meat value should be sometimes substituted for meat. They have prepared recipes according to which a savoury meal can be prepared for a hundred children at a cost of a penny a head. These meals require from two to three hours to cook, but are so simple in preparation that they demand no special culinary skill. If children will eat and can enjoy these foods they will not only be well fed, but they will have acquired a taste for wholesome and economical food which will enable them to live well on an income on which the consumers of more costly but not more nourishing food would come near to starving.

